# Dual‐Center Repeatability, Consistency, and Comparison of 3D Phase‐Resolved Functional (PREFUL) Ventilation MRI at 3 and 1.5 T in Healthy Volunteers

**DOI:** 10.1002/nbm.70101

**Published:** 2025-07-15

**Authors:** Filip Klimeš, Chuan T. Foo, Marcel Gutberlet, Andreas Voskrebenzev, Richard McIntyre, Marius M. Wernz, Norman Kornemann, Rimma Kondrashova, Robin A. Müller, Robert Grimm, Frank Wacker, Francis Thien, Jens Vogel‐Claussen

**Affiliations:** ^1^ Institute of Diagnostic and Interventional Radiology Hannover Medical School Hannover Germany; ^2^ Biomedical Research in Endstage and Obstructive Lung Disease Hannover (BREATH) German Center for Lung Research (DZL) Hannover Germany; ^3^ Department of Respiratory Medicine Eastern Health Melbourne Victoria Australia; ^4^ Faculty of Medicine, Nursing and Health Sciences Monash University Melbourne Victoria Australia; ^5^ Monash Biomedical Imaging Monash University Melbourne Victoria Australia; ^6^ Monash Imaging Department Monash Health Melbourne Victoria Australia; ^7^ Research & Clinical Translation, Magnetic Resonance Siemens Healthineers AG Erlangen Germany

**Keywords:** 3D PREFUL, consistency, field strength, lung MRI, repeatability, ventilation imaging

## Abstract

3D phase‐resolved functional lung (PREFUL) MRI offers lung ventilation assessment without contrast and in free breathing. However, there are limited data on its repeatability and intercenter consistency. This study aimed to assess the repeatability and intercenter consistency of 3D PREFUL MRI at two centers using both 3‐ and 1.5‐T field strengths. Fifty healthy volunteers (35 female, 70%; median age 25.5 [interquartile range 22.3–29.0 years]) participated in this prospective study. A golden‐angle stack‐of‐stars spoiled gradient echo acquisition was used for 3D PREFUL MRI. To assess for repeatability, 3D PREFUL MRI was performed twice with a short interval between scans at each center. At Center 1, scans were performed at 3 and 1.5 T. At Center 2, scans were performed at 3 T. Intercenter consistency of 3D PREFUL MRI was assessed between centers at 3 T. Performance of 3D PREFUL MRI at 3 and 1.5 T was compared at Center 1. Bland–Altman analysis, coefficient of variation, intraclass correlation coefficient, and Cohen's *d* were used for the repeatability assessment. Similarly, interfield strength and intercenter differences were quantified by Bland–Altman analysis. In all comparisons, paired Wilcoxon signed‐rank test was used to examine differences. 3D PREFUL MRI parameters showed no significant differences between repeated measurements across three scanners, except for mean regional ventilation (RVent) at Center 2 (*p* = 0.0046). Apart from mean RVent during the second measurement (*p* = 0.03) and tidal volume during both measurements (both *p* < 0.02), no significant differences were found between 3D PREFUL MRI parameters at 3 T across both centers. Output parameters were significantly different when images were acquired using 3 T compared to 1.5 T (all *p* < 0.0093). 3D PREFUL MRI ventilation‐weighted parameters showed fair to excellent repeatability and consistent intercenter results. Given the significant differences in outputs acquired using scanners of different field strengths, multicenter studies should be conducted with scanners of the same field strength.

## Graphical abstract

3D PREFUL MRI offers a ventilation‐weighted assessment without contrast and during free breathing. This study evaluated its repeatability and consistency across 1.5‐ and 3‐T scanners at two centers. Results showed high repeatability and consistency, but significant differences between 1.5 and 3 T emphasize the need for consistent field strengths in multicenter studies. These findings support 3D PREFUL MRI as a reliable, noninvasive tool for lung ventilation assessment.

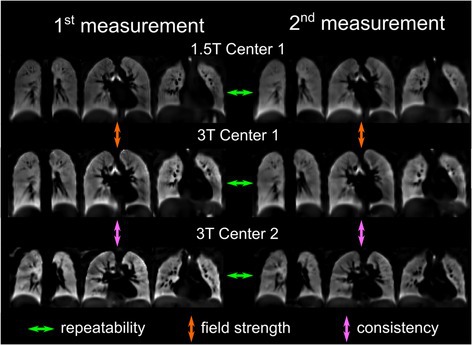

**Abbreviations**
ANTs: advanced normalization tools
BART: Berkeley Advanced Reconstruction Toolbox
BMI: body mass index
CoV: coefficient of variation
FD: Fourier decomposition
FER: forced expiratory ratio
FEV_1_
: forced expiratory volume in 1 s
FVC: forced vital capacity
FVL‐CM: flow‐volume‐loop correlation metric
GOREG: group‐oriented registration
ICC: intraclass correlation coefficient
IQR: interquartile range
PFT: pulmonary function test
PREFUL: phase‐resolved functional lung
RVent: regional ventilation
UTE: ultrashort echo time
VVP: ventilated volume percentage

## 1 Introduction

In recent years, several free‐breathing pulmonary imaging techniques based on Fourier decomposition (FD) [1, 2] have gained interest for functional assessment of the lung. FD MRI enables assessment of ventilation and perfusion‐weighted maps by analyzing the temporal evolution of dynamic pulmonary MRI timeseries acquired without contrast agents or breath‐holding. Compared to nuclear medicine methods or CT, FD MRI‐based methods avoid harmful ionizing radiation, and unlike hyperpolarized gas MRI techniques, they do not require inhalation of a contrast agent or specialized equipment. To date, most FD MRI research has focused on 2D sequences [3, 4, 5], as they provide analysis of both perfusion and ventilation. However, recent advancements have led to the development of 3D techniques aimed at improving lung coverage [6, 7, 8, 9]. Currently, 3D methods are primarily limited to ventilation‐weighted information due to technical constraints, as the blood inflow effect is eliminated by the use of nonselective hard RF pulses in the MR sequence design. One of these methods, 3D phase‐resolved functional lung (PREFUL) MRI, offers functional ventilation assessment of the whole lung using a pseudo‐3D stack‐of‐stars acquisition [10]. Comparison of 3D PREFUL MRI to direct measurements of ventilation using ^19^F imaging [11], as well as ^129^Xe [12, 13] imaging, has showed promising results, particularly in terms of global agreement. Ventilation parameters derived by 3D PREFUL MRI have also demonstrated good repeatability in a study involving 53 healthy subjects and 13 patients with chronic obstructive pulmonary disease at 1.5 T [14]. As all of these studies were conducted at a single center, the intercenter consistency of 3D PREFUL MRI remains unclear. Additionally, there is limited evidence comparing the performance of 3D PREFUL MRI between 1.5‐ and 3‐T systems, with the latter commonly used in clinical settings. When compared to 1.5 T, the higher field strength of 3 T offers a higher achievable signal‐to‐noise ratio and faster imaging in many clinical applications [15]. However, in the lung, the theoretical advantage of 3 T might be outweighed by the increased effect of magnetic susceptibility, which decreases the short T2* relaxation time even further [16]. Understanding the variability between scanners at different institutions and field strengths is an essential step prior to the wider dissemination of 3D PREFUL MRI to other research centers.

Therefore, in this study, we aim to (i) assess the repeatability and intercenter consistency of 3D PREFUL MRI across two centers and (ii) evaluate its performance at 3 T compared to 1.5 T at a single center.

## 2 Methods

### 2.1 Study Population

A total of 50 healthy volunteers were recruited across both centers, including 24 (10 male, 14 female, median age: 25.5 years; interquartile range [IQR]: 23.8–28.0) from Center 1 and 26 (5 male, 21 female, median age: 25.5 years; IQR: 22.0–33.0) from Center 2 (Table 1a).

**TABLE 1:** (a) Study demographics and pulmonary function testing data for all study subjects at both included centers. (b) Parameter details of the stack‐of‐stars sequence with the golden‐angle increment for all scanners included.

a.	Center 1	Center 2	*p* ^a^
Age (years)	25.5 (23.8–28.0)	25.5 (22.0–33.0)	0.98
Sex (–)	14F/10M	21F/5M	0.08
BMI (kg/m^2^)	22.0 (20.6–23.7)	23.0 (21.3–25.2)	0.15
FEV_1_ (% predicted)	99.2 (93.2–102.9)	98.0 (90.0–102.0)	0.65
FVC (% predicted)	101.1 (95.7–105.3)	101.5 (97.3–105.8)	0.93
FER (%)	80.9 (77.4–87.8)	84.8 (78.5–87.6)	0.53

b.	Center 1—1.5 T	Center 1—3 T	Center 2—3 T
Scanner manufacturer name	MAGNETOM Aera	MAGNETOM Vida	MAGNETOM Skyra
Slew rate (T/m/s)	200	200	200
Maximum gradient amplitude (mT/m)	45	60	45
Transmit coil	Body, 1 channel	BioMatrix Body, 2 channels	Body, 2 channels
Receive coil	Spine, 32 channels	BioMatrix Spine RS, 32 channels	Spine, 32 channels
TE (ms)	0.8	0.77	0.79
TR (ms)	1.9	1.9	1.9
FOV (cm^3^)	50 × 50 × 17.2 − 25.0	50 × 50 × 17.2 − 25.0	50 × 50 × 15.6 − 28.1
Matrix size (–)	128 × 128 × 44 − 64	128 × 128 × 44–64	128 × 128 × 40 − 72
Slice thickness (mm)	3.9	3.9	3.9
Bandwidth (Hz/px)	1560	1560	1560
Flip angle (°)	4	3	3
TA (min)	8	8	8

*Note:* Unless otherwise stated, values are medians with interquartile ranges in parentheses.

Abbreviations: BMI, body mass index; F, female; FER, forced expiratory ratio; FEV_1_, forced expiratory volume in 1 s; FOV, field of view; FVC, forced vital capacity; M, male; TA, acquisition time; TE, echo time; TR, repetition time.

^a^ Wilcoxon rank‐sum test was used for all comparisons, except for sex, where a chi‐square test was applied. *p* value < 0.05 was considered significant.

Eligible subjects had to be aged 18 years and above, have no pre‐existing medical history, and be nonsmokers. Subjects aged less than 18 years, or who were unable to undergo MRI scanning, or provide written informed consent were excluded. The study was approved by the institutional review board of each center. All subjects provided written informed consent.

### 2.2 Image Acquisition and Protocol

**FIGURE 1:**
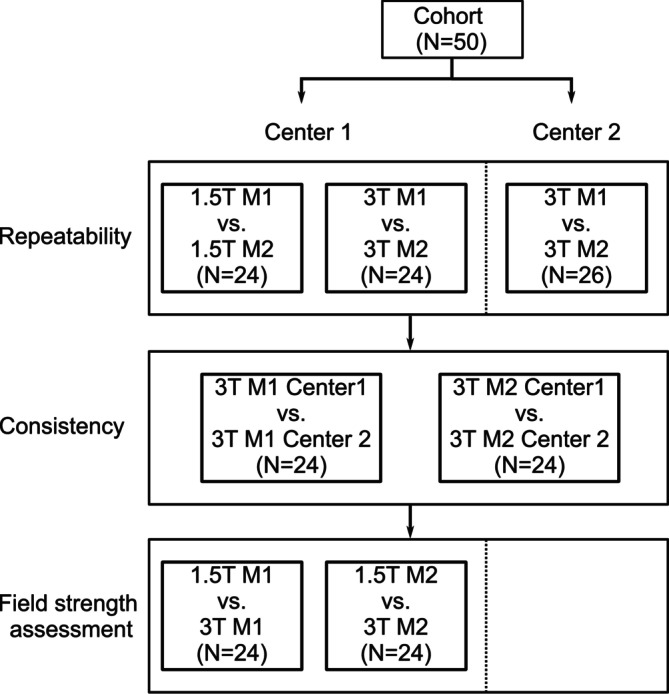
Study flowchart. At each MR scanner 3D PREFUL MRI measurements were repeated. M, 3D PREFUL MRI measurement.

At both centers, MR images were acquired using a 3D spoiled gradient echo sequence with stack‐of‐stars trajectory over 8 min with subjects free breathing. Detailed sequence parameters are listed in Table 1b. Imaging was performed on a 1.5‐ (MAGNETOM Aera, Siemens Healthineers, Forchheim, Germany) and 3‐T scanner (MAGNETOM Vida, Siemens Healthineers, Forchheim, Germany) at Center 1 and only on a 3‐T scanner (MAGNETOM Skyra, Siemens Healthineers, Forchheim, Germany) at Center 2. For each scanner, two MR acquisitions (first and second measurements) were performed after a minimum of 10 min outside the scanner. During the break, subjects were instructed to relax, without inducing any physiological changes. No additional preparation was required between scans. All acquisitions were performed with the same scan parameters, positioning protocol, and coil setup to ensure consistency across repeated measurements. Repeatability was assessed for all three scanners. Intercenter consistency was evaluated at 3 T between both centers. Field strength comparison between 3 and 1.5 T was performed at Center 1. The MR exams for the field strength comparison were performed on the same day whenever scanner availability permitted. A flow chart of the study protocol is shown in Figure 1.

### 2.3 Image Reconstruction and Postprocessing

Image reconstruction and postprocessing were performed at Center 1 using the 3D PREFUL pipeline as previously described [17]. Raw data acquired at Center 2 were transferred to Center 1 for this purpose. Briefly, all data were gradient delay corrected using a radial intersection gradient delay estimation method [18] implemented in the Berkeley Advanced Reconstruction Toolbox (BART) [19]. For each subject and measurement, the gradient delay–corrected acquisitions were sorted into respiratory bins (*N* bin range: 27–51), with each bin containing at least 100 radial projections and incorporating 20% view sharing from neighboring bins. The number of respiratory bins varied between subjects due to anatomical differences that led to different numbers of acquired slices. Given the same acquisition time for all study subjects, varying amounts of radial projections per slice were available for image reconstruction. The parallel imaging and compressed sensing image reconstruction was performed using BART [19], leveraging sparsity in both the image and temporal domains by applying L2 norm (*λ* = 0.1) and total variation (*λ* = 0.0001) in the spatial domain and total variation regularization (*λ* = 0.01) in the temporal domain. To enable voxel‐wise functional analysis of the temporally resolved data, a group‐oriented registration approach [20] was applied using the Forsberg toolbox [21] to align all respiratory phases to the end‐inspiratory phase image. Additionally, the morphological images of the second measurement were spatially registered to the end‐inspiratory image used for the spatial alignment of the first measurement.

For all 3D PREFUL MRI measurements, regional ventilation (RVent) maps were computed for each respiratory phase [22], and the RVent cycle was analyzed by flow‐volume loops (FVLs) [23]. FVL cross‐correlation metric (FVL‐CM) maps, which depict the homogeneity of RVent dynamics throughout the whole ventilation cycle, were also derived by cross‐correlating voxel‐wise FVL to a reference FVL [24]. To account for any potential bias caused by differences in tidal volumes across 3D PREFUL measurements, the tidal volume of each measurement was estimated using deep learning–based lung parenchyma segmentation [25] as the difference in lung volume between end‐inspiratory and end‐expiratory morphological images. Breathing frequency (in breaths per minute) was calculated from the low spatial‐resolution images (matrix size: 32 × 32, nominal temporal resolution: 100 ms) which were used to extract the gating signal for the image reconstruction.

For the image analysis, the lung parenchyma mask was segmented in the end‐inspiratory image using a deep‐learning algorithm [25] with large vessels extracted using the vessel recognition algorithm proposed by Wernz et al. [26].

### 2.4 Pulmonary Functional Testing (PFT)

All subjects performed spirometry at their respective centers. All tests were conducted in accordance with European Respiratory Society/American Thoracic Society standards [27]. Reference values for spirometry were taken from the 2012 Global Lung Initiative equations [28].

### 2.5 Statistical Analysis

The means and coefficients of variation (CoVs) for RVent within the lung parenchyma mask, as well as the means of FVL‐CM within the same mask, were calculated. Subsequently, the ventilated volume percentage (VVP_RVent_ and VVP_FVL‐CM_) values were calculated using published thresholds [24, 29]. An overview of all 3D PREFUL MRI‐derived parameters and their clinical rationale is provided in Table 2.

**TABLE 2:** Overview of 3D PREFUL MRI‐derived ventilation parameters.

Parameter name	Metric	Abbreviation	Units	Definition	Clinical justification
Regional ventilation	Mean	Mean RVent	mL/mL	Mean of voxel‐wise values calculated as RVent = *S*_Ref_/*S*_Insp_ − *S*_Ref_/*S*_Exp_ [22].	Static ventilation marker, quantifies voxel‐wise ventilation. Drawback: Depends on breathing frequency and tidal volume.
	Coefficient of variation	CoV RVent	%	Ratio of standard deviation to the mean of RVent values.	Reflects heterogeneity of static ventilation.
	Ventilated volume percentage	VVP_RVent_	%	Proportion of voxels with RVent above 40% of the 90th percentile threshold [29].	Indicates ventilated lung proportion, correlated strongly to ventilation defect percentage (VDP) derived by ^129^Xe [12, 29] and ^19^F imaging [11].
Flow‐volume‐loop cross‐correlation metric–based ventilation	Mean	Mean FVL‐CM	a.u.	Mean of voxel‐wise cross‐correlations between each voxel's FVL and a reference FVL [3].	Captures abnormalities in local ventilation dynamics and significantly discriminates between the presence and absence of chronic lung allograft dysfunction [24].
	Coefficient of variation	CoV FVL‐CM	%	Ratio of standard deviation to the mean of FVL‐CM values.	Indicates variation in ventilation dynamics throughout the whole ventilation cycle.
	Ventilated volume percentage	VVP_FVL‐CM_	%	Proportion of voxels with FVL‐CM values above a fixed threshold of 0.9 [24].	Indicates ventilated lung proportion, has been shown to be a predictor of poorer survival in patients who have undergone lung transplantation [30].

Abbreviations: CoV, coefficient of variation (assessing the heterogeneity of the ventilation maps); FVL‐CM, flow‐volume loop cross‐correlation metric; ICC, intraclass correlation coefficient; Mean bias, mean difference derived from Bland–Altman analysis; RVent, regional ventilation; *S*_Exp_, MR signal of expiratory phase; *S*_Insp_, MR signal of inspiratory phase; *S*_Ref_, MR signal of reference phase used for image registration; VVP_FVL‐CM_, ventilated volume percentage based on FVL‐CM; VVP_RVent_, ventilated volume percentage based on RVent.

Given the small sample size, nonparametric tests were used as a conservative approach for all statistical comparisons. To compute the repeatability of 3D PREFUL MRI at each scanner, differences between ventilation parameters (mean RVent, CoV RVent, mean FVL‐CM, VVP_RVent_, VVP_FVL‐CM_, tidal volume, and breathing frequency) derived from the first and second 3D PREFUL MRI measurements were tested for significance using paired Wilcoxon signed‐rank test. The agreement between both measurements was assessed by Bland–Altman analysis, median CoV, and intraclass correlation coefficient (ICC) analysis. ICC values were evaluated as the degree of absolute agreement among both measurements using the two‐way random effects model [31] and interpreted using the scale published by Viera and Garrett [32]. Cohen's *d* was calculated to quantify the effect size in assessing the repeatability of paired measurements. The magnitude of the effect size was interpreted using the scale proposed by Sawilowsky [33], where effect sizes are classified as very small (0.01), small (0.2), medium (0.5), large (0.8), very large (1.2), and huge (2.0). To assess the regional (voxel‐wise) agreement of the VV maps (VV_RVent_ and VV_FVL‐CM_), a spatial overlap metric was calculated. The spatial overlap was defined as the percentage of matching voxels labeled as either ventilated (healthy) or nonventilation (defect) between two VV maps and was calculated as

$$\text{Overlap}=\left(\frac{2\left(n_{\mathrm{vv}}+n_{\mathrm{dd}}\right)}{\left(n_{v1}+n_{v2}+n_{d1}+n_{d2}\right)}\right)*100.$$

Here, *n*_*vv*_ is the number of voxels labeled as ventilated in both maps (healthy), and *n*_*dd*_ is the number labeled as nonventilated (defect) in both maps. The terms *n*_*v*1_ and *n*_*v*2_ refer to the number of voxels labeled as ventilated in VV Map 1 and VV Map 2, respectively, while *n*_*d*1_ and *n*_*d*2_ denote the number of voxels labeled as nonventilated (defect) in VV Map 1 and VV Map 2.

In assessing consistency between centers, subjects were age‐matched and ventilation parameters derived at 3 T were tested for differences using paired Wilcoxon signed‐rank test.

In the field strength comparison at Center 1, ventilation parameters derived at 3 T were compared to parameters derived at 1.5 T using similar tests as described in the repeatability comparison above. In addition to Bland–Altman analysis, the mean differences were also quantified by percentage change.

All the statistical tests were performed using MATLAB (R2022b, The Mathworks Inc., Natick, MA, USA) and results were deemed significant when *p* < 0.05.

## 3 Results

All study subjects successfully completed both MRI sessions and pulmonary function tests without any reported issues. All subjects had normal spirometry. No significant differences were observed between subjects at the two centers in terms of age, sex, body mass index, or spirometry (all *p* ≥ 0.08, Table 1a). Medians and IQR of 3D PREFUL ventilation parameters and calculated tidal volumes derived for all measurements are presented in Table 3.

**TABLE 3:** Repeatability comparison of 3D PREFUL MRI ventilation parameters derived at both centers.

a. Center 1—1.5 T (*N* = 24)	First measurement	Second measurement	Mean bias	*p* ^a^	CoV (%)	ICC (–)	Cohen's *d* (–)
Mean RVent (mL/mL)	0.17 (0.12–0.23)	0.17 (0.11–0.21)	0.02	0.12	11.42	0.88	0.39
CoV RVent (%)	31.57 (29.14–35.14)	33.20 (28.83–37.48)	−0.94	0.20	5.26	0.85	−0.27
Mean FVL‐CM (–)	0.99 (0.98–0.99)	0.98 (0.97–0.99)	0.004	0.65	0.31	0.37	0.26
VVP_RVent_ (%)	95.70 (94.35–98.06)	95.81 (93.16–98.17)	0.49	0.82	0.61	0.63	0.18
VVP_FVL‐CM_ (%)	98.93 (96.42–99.73)	97.78 (94.48–99.64)	1.25	0.22	0.81	0.33	0.25
Tidal volume (mL)	390.44 (318.72–690.72)	433.71 (303.31–649.06)	40.37	0.18	12.51	0.86	0.35
Breathing frequency (breaths/min)	14.42 (9.94–18.09)	13.87 (9.85–16.77)	1.09	0.22	8.93	0.58	0.23

b. Center 1—3 T (*N* = 24)	First measurement	Second measurement	Mean bias	*p* value^a^	CoV (%)	ICC (–)	Cohen's *d* (–)
Mean RVent (mL/mL)	0.20 (0.17–0.26)	0.19 (0.16–0.26)	0.005	0.63	7.17	0.83	0.11
CoV RVent (%)	37.11 (31.03–43.41)	35.05 (32.70–42.21)	1.44	0.22	7.30	0.80	0.25
Mean FVL‐CM (–)	0.98 (0.95–0.99)	0.97 (0.95–0.98)	0.001	0.71	0.96	0.76	0.03
VVP_RVent_ (%)	92.86 (87.49–96.20)	93.44 (89.40–96.19)	−1.11	0.39	0.65	0.62	−0.16
VVP_FVL‐CM_ (%)	96.03 (88.62–98.91)	94.61 (86.21–97.22)	0.57	0.67	2.23	0.77	0.10
Tidal volume (mL)	419.94 (329.82–521.76)	388.44 (298.93–534.33)	24.43	0.21	11.05	0.65	0.17
Breathing frequency (breaths/min)	15.06 (11.30–17.60)	15.19 (10.02–17.53)	0.03	0.71	6.97	0.75	0.01

c. Center 2—3 T (*N* = 26)	First measurement	Second measurement	Mean bias	*p* value^a^	CoV (%)	ICC (–)	Cohen's *d* (–)
Mean RVent (mL/mL)	0.19 (0.15–0.25)	0.16 (0.12–0.20)	0.03	0.0046*	10.96	0.74	0.70
CoV RVent (%)	34.86 (30.10–41.22)	34.23 (29.18–41.80)	−0.19	0.71	6.88	0.88	−0.04
Mean FVL‐CM (–)	0.98 (0.97–0.99)	0.98 (0.97–0.99)	−0.001	0.93	0.42	0.66	−0.04
VVP_RVent_ (%)	93.70 (88.42–96.38)	93.63 (87.66–96.93)	0.05	0.73	1.12	0.90	0.02
VVP_FVL‐CM_ (%)	97.73 (94.89–99.11)	97.88 (92.70–99.46)	−0.56	0.55	0.68	0.48	−0.12
Tidal volume (mL)	315.16 (212.83–438.51)	251.62 (192.27–347.00)	39.55	0.14	9.97	0.85	0.42
Breathing frequency (breaths/min)	12.26 (9.19–14.23)	12.78 (11.57–16.52)	−1.10	0.22	8.58	0.53	−0.31

*Note:* Values of first and second 3D PREFUL measurements are expressed as a median with interquartile range in parentheses.

Abbreviations: CoV, coefficient of variation; FVL‐CM, flow‐volume loop cross‐correlation metric; ICC, intraclass correlation coefficient; Mean bias, mean difference derived from Bland–Altman analysis; RVent, regional ventilation; VVP_FVL‐CM_, ventilated volume percentage based on FVL‐CM; VVP_RVent_, ventilated volume percentage based on RVent.

^a^ Two‐sided paired Wilcoxon signed‐rank test, *p* value < 0.05 was considered significant; significantly different measurements are marked with *.

### 3.1 Repeatability

**FIGURE 2:**
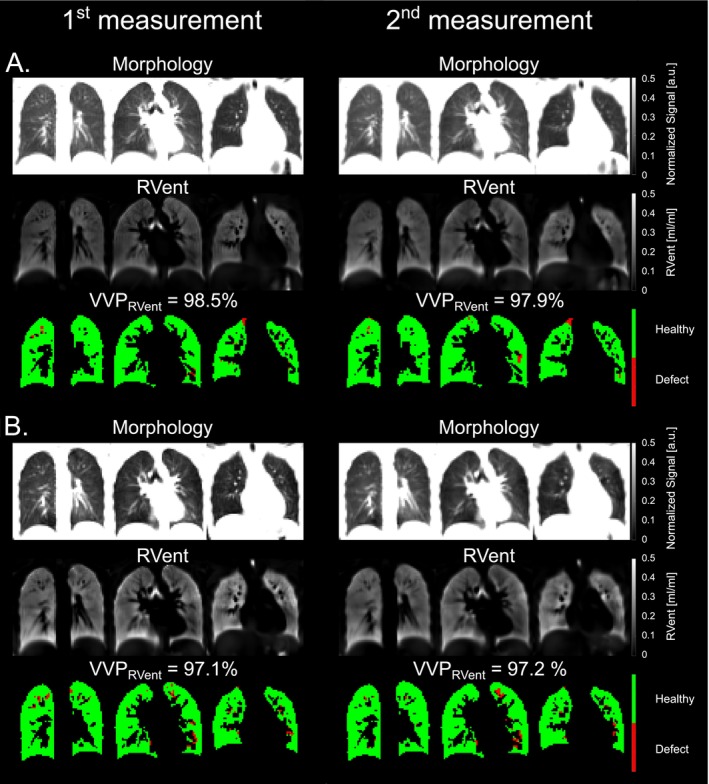
Repeatability comparison of static 3D PREFUL MRI parameters at Center 1 at both field strengths. Representative morphological, static regional ventilation (RVent) maps and corresponding binary ventilated volume maps (A—1.5 T Center 1, B—3 T Center 1) for a healthy volunteer (26‐year‐old female, FEV_1_ = 99% pred.) of the first (left) and second (right) 3D PREFUL measurements. Mean RVent of the first measurement for the whole lung volume was 0.15 mL/mL (coefficient of variation [CoV] of 28%) at 1.5 T and 0.22 mL/mL (CoV of 31%) at 3 T. Mean RVent of the second measurement for the whole lung volume was 0.17 mL/mL (CoV of 31%) at 1.5 T and 0.20 mL/mL (CoV of 30%) at 3 T. VVP_RVent_, ventilated volume percentage based on RVent parameter.

**FIGURE 3:**
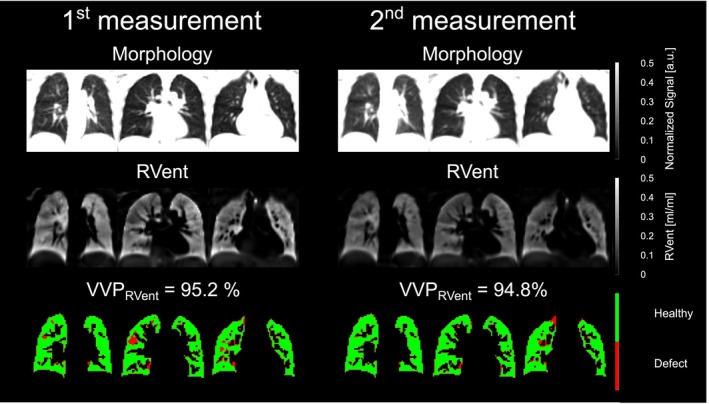
Repeatability comparison of static 3D PREFUL MRI parameters at Center 2. Representative morphological, regional ventilation (RVent) maps and corresponding binary ventilation volume maps for a healthy volunteer (33‐year‐old female, FEV_1_ = 111% pred.) from Center 2 of the first (left) and second (right) 3D PREFUL measurements. Mean RVent of the first measurement for the whole lung volume was 0.25 mL/mL (coefficient of variation [CoV] of 30%) 1.5 T and 0.18 mL/mL (CoV of 31%) for the second measurement, respectively. The mean RVent differences were pronounced in the study population at Center 2 suggesting for habituation effects, as the subjects were more relaxed during second 3D PREFUL MRI measurement which resulted in significantly lower mean RVent parameter.

**FIGURE 4:**
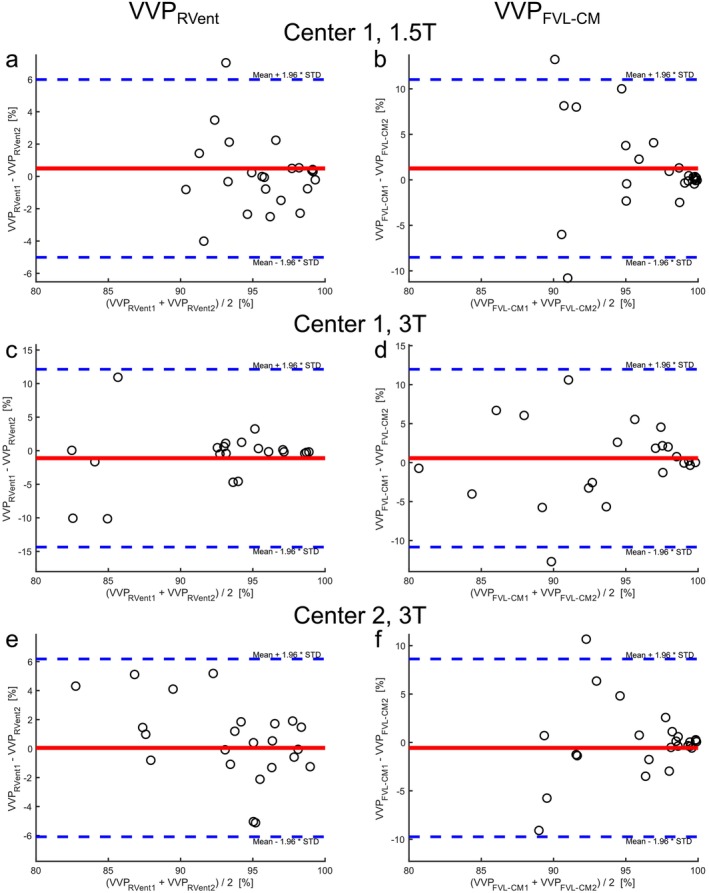
Repeatability of 3D PREFUL MRI VVP measurements analyzed by Bland–Altman plots. (a) VVP_RVent_ measurement at 1.5 T and Center 1 (mean bias of 0.49% with 95% limits of agreement −5.01% to 5.99%), (b) VVP_FVL‐CM_ measurement at 1.5 T and Center 1 (mean bias of 1.25% with 95% limits of agreement −8.51% to 11.02%), (c) VVP_RVent_ measurement at 3 T and Center 1 (mean bias of −1.11% with 95% limits of agreement −14.35% to 12.13%), (d) VVP_FVL‐CM_ measurement at 3 T and Center 1 (mean bias of 0.57% with 95% limits of agreement −10.84% to 11.97), (e) VVP_RVent_ measurement at 3 T and Center 2 (mean bias of 0.05% with 95% limits of agreement −6.28% to 6.18%), and (f) VVP_FVL‐CM_ measurement at 3 T and Center 2 (mean bias of −0.56% with 95% limits of agreement −10.13% to 8.62%).

The repeatability assessments are shown in Table 3: (a) for Center 1 with the 1.5‐T scanner, (b) for Center 1 with the 3‐T scanner, and (c) for Center 2 with the 3‐T scanner. Visual comparisons between both measurements for each scanner are displayed in Figure 2 (Center 1) and Figure 3 (Center 2). No significant bias was observed between the first and second measurements at both centers, except for the mean of the RVent parameter derived at Center 2, which showed lower ventilation values in the second measurement (mean bias of 0.03 mL/mL, *p* = 0.0046, Table 3c). Exemplary Bland–Altman plots for VVP_RVent_ and VVP_FVL‐CM_ parameters are presented in Figure 4.

Median CoV between the two measurements of all calculated 3D PREFUL parameters was less than 11.4% at both centers and both field strengths (Table 3).

The ICC values ranged from 0.33 to 0.90, with most parameters showing substantial to almost perfect (ICC > 0.61) repeatability agreement. A fair agreement (ICC between 0.21 and 0.4) was observed for mean FVL‐CM and VVP_FVL‐CM_ parameters for 1.5‐T scanner at Center 1.

Across all scanners the absolute Cohen's *d* values were lowest for mean FVL‐CM as well as for VVP_RVent_ and VVP_FVL‐CM_ (all |values| < 0.25), indicating a small effect size for the majority of 3D PREFUL MRI parameters. On the other hand, the highest Cohen's *d* values were observed for mean RVent at Center 1 (1.5 T) and Center 2 (3 T), with values ranging from 0.39 to 0.70.

In the regional comparison, the median spatial overlap at Center 1 with the 1.5‐T scanner was 95.5% (93.3%–97.8%) for VV_RVent_ and 97.2% (91.5%–99.5%) for VV_FVL‐CM_. At the same center using the 3‐T scanner, the overlap was 92.2% (85.5%–95.6%) for VV_RVent_ and 90.9% (82.8%–96.9%) for VV_FVL‐CM_. At Center 2 with the 3‐T scanner, the median overlap was 94.1% (90.8%–96.5%) for VV_RVent_ and 96.9% (89.3%–98.8%) for VV_FVL‐CM_.

### 3.2 Consistency Between Centers Using Independent Cohorts at 3 T

For the intercenter consistency analyses, comparisons were made between subjects scanned at 3 T between Centers 1 and 2. As these represented two separate cohorts, an age‐matching comparison was performed, which resulted in the exclusion of Subjects #1 (F, 49 years old) and #5 (M, 62 years old) from Center 2. The median age difference after age matching was 2 years (IQR: 1–4).

No significant bias was observed between the age‐matched 3D PREFUL measurements (Table 4, all *p* > 0.06), except for the mean RVent in the second measurement (mean bias of 0.05 mL/mL towards higher values at Center 1, *p* = 0.0278).

**TABLE 4:** Consistency comparison of 3D PREFUL MRI ventilation parameters across centers. The subjects were matched according to their age. A positive bias (+) indicates that the values at Center 1 were higher than at Center 2, while a negative bias (–) indicates that the values at Center 2 were higher than at Center 1.

Center 1 vs. Center 2, *N* = 24, 3 T vs. 3 T	Mean bias—First measurement	*p* ^a^	Mean bias—Second measurement	*p* ^a^
Mean RVent (mL/mL)	0.02	0.42	0.05	0.0278*
CoV RVent (%)	1.83	0.39	0.16	1.00
Mean FVL‐CM (–)	−0.01	0.33	−0.01	0.07
VVP_RVent_ (%)	−1.05	0.48	0.10	0.93
VVP_FVL‐CM_ (%)	−3.09	0.18	−4.27	0.06
Tidal volume (mL)	128.32	0.0177*	142.00	0.0061*
Breathing frequency (breaths/min)	2.55	0.10	1.35	0.38

Abbreviations: CoV, coefficient of variation (assessing the heterogeneity of the ventilation maps); FVL‐CM, flow‐volume loop cross‐correlation metric; Mean bias, mean difference derived from Bland–Altman analysis; RVent, regional ventilation; VVP_FVL‐CM_, ventilated volume percentage based on FVL‐CM; VVP_RVent_, ventilated volume percentage based on RVent.

^a^ Two‐sided paired Wilcoxon signed‐rank test, *p* value < 0.05 was considered significant; significantly different measurements are marked with *.

The mean tidal volumes for Center 1 were 419.94 mL for the first measurement and 388.44 mL for the second 3D PREFUL MRI measurement. Similarly, for Center 2, the mean tidal volumes were 316.51 mL for the first and 278.40 mL for the second 3D PREFUL MRI measurement. Although study subjects at Center 1 showed significant increases in tidal volumes for both measurements (mean bias ≥ 128.32 mL, both *p* ≤ 0.0177), these did not result in any differences in the VVP parameters (all *p* ≥ 0.06). No significant differences between breathing frequencies were seen (both *p* > 0.10).

**FIGURE 5:**
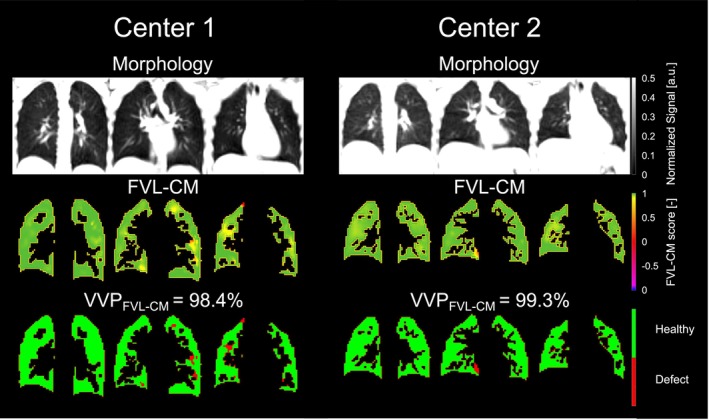
Comparison of age‐matched volunteers among both recruiting centers. Representative morphological, dynamic flow‐volume‐loop correlation metric (FVL‐CM) maps and corresponding binary ventilated volume maps for a healthy volunteer (22‐year‐old female, FEV_1_ = 105% pred.) from Center 1 (left) and a healthy volunteer (20‐year‐old female, FEV_1_ = 99% pred.) from Center 2 (right) 3D PREFUL measurement. Mean FVL‐CM at Center 1 for the whole lung volume was 0.99 and 0.99 for Center 2, respectively. There were no statistical differences among centers in comparisons of 3D PREFUL parameters derived from dynamic FVL‐CM parameter and in both ventilation volume percentage (VVP) parameters. Please note difference in total lung volume between both subjects, this was statistically significant between both groups.

In Figure 5 a visual comparison of two age‐matched volunteers and their 3D PREFUL ventilation‐weighted maps is depicted.

### 3.3 Field Strength Comparison at Center 1

**FIGURE 6:**
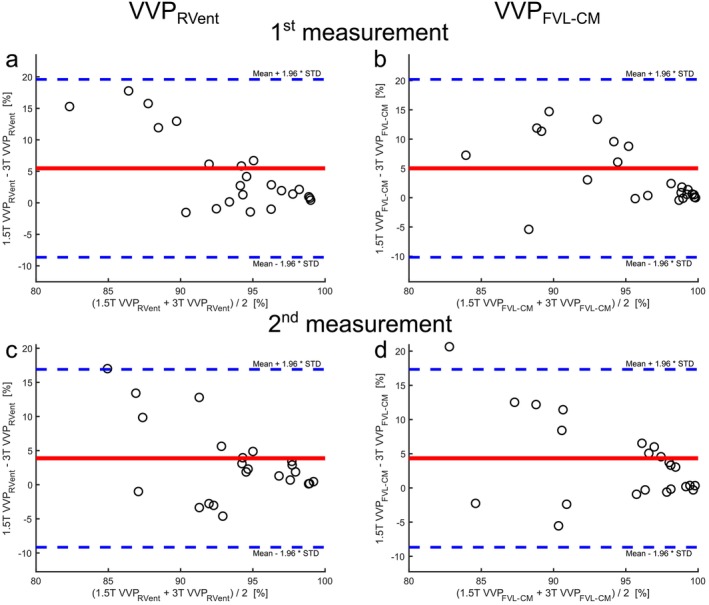
Comparison of 3D PREFUL MRI VVP measurements across different field strengths at Center 1 analyzed by Bland–Altman plots. (a) First VVP_RVent_ measurement (mean bias of 5.48% with 95% limits of agreement −8.64% to 19.60%), (b) first VVP_FVL‐CM_ (mean bias of 5.02% with 95% limits of agreement −4.71% to 14.74%), (c) second VVP_RVent_ measurement (mean bias of 3.87% with 95% limits of agreement −9.16% to 16.91%), and (d) second VVP_FVL‐CM_ (mean bias of 4.33% with 95% limits of agreement −10.85% to 19.52).

Table 5 summarizes the results of the field strength comparison between 3 and 1.5 T at Center 1. For the first measurement, significant differences were observed in all 3D PREFUL parameters except for tidal volume (*p* = 0.29) and breathing frequency (*p* = 0.56) when images were acquired on a 3 T compared to a 1.5 T (all *p* < 0.0043). Similar results were obtained for the second measurement (all *p* < 0.0093). The respective percentage changes are listed in Table 5. The Bland–Altman analysis for VVP_RVent_ and VVP_FVL‐CM_ is shown in Figure 6.

**TABLE 5:** Comparison of 3D PREFUL MRI ventilation parameters across different field strength at Center 1. A positive bias (+) indicates that the 1.5‐T values were higher than 3‐T values, while a negative bias (–) indicates that the 3‐T values were higher than the 1.5‐T values.

Center 1, *N* = 24, 1.5 T vs. 3 T	Mean bias—First measurement (% change)	*p* ^a^	Cohen's *d* (–)	Mean bias—Second measurement (% change)	*p* ^a^	Cohen's *d* (–)
Mean RVent (mL/mL)	−0.03 (+18%)	0.0043*	−0.71	−0.04 (+12%)	0.0006*	−0.97
CoV RVent (%)	−6.06 (+18%)	0.0005*	−0.90	−3.67 (+6%)	0.0093*	−0.64
Mean FVL‐CM (–)	0.02 (−1%)	0.0002*	0.73	0.01 (−1%)	0.0017*	0.77
VVP_RVent_ (%)	5.48 (−3%)	0.0005*	0.76	3.87 (−2%)	0.0086*	0.58
VVP_FVL‐CM_ (%)	5.02 (−3%)	0.0005*	0.65	4.33 (−3%)	0.0086*	0.65
Tidal volume (mL)	50.70 (+8%)	0.29	0.37	34.75 (−10%)	0.23	0.28
Breathing frequency (breaths/min)	−0.08 (+4%)	0.56	−0.02	−1.15 (+10%)	0.048*	−0.24

Abbreviations: CoV, coefficient of variation (assessing the heterogeneity of the ventilation maps); FVL‐CM, flow‐volume loop cross‐correlation metric; Mean bias, mean difference derived from Bland–Altman analysis; RVent, regional ventilation; VVP_FVL‐CM_, ventilated volume percentage based on FVL‐CM; VVP_RVent_, ventilated volume percentage based on RVent.

^a^ Two‐sided paired Wilcoxon signed‐rank test, *p* value < 0.05 was considered significant; significantly different measurements are marked with *.

Specifically, RVent values (mean and CoV) were significantly increased at 3 T (all *p* ≤ 0.0093) leading also to significantly lower VVP_RVent_ values (mean bias of 5.5% and 3.9% for first and second measurement, respectively, both *p* ≤ 0.0086).

Also, a significant decrease of mean FVL‐CM values was observed at 3 T compared to 1.5 T (both *p* ≤ 0.0017, with mean biases of 0.02 and 0.01 for first and second measurements). These pronounced variances led to significantly decreased VVP_FVL‐CM_ for both 3D PREFUL measurements at the 3‐T scanner (mean bias of 5.0% and 4.3%, respectively, both *p* ≤ 0.0086).

Most of the Cohen's *d* values fell within the moderate to large effect size range (see Table 5), indicating that the bias observed in the Bland–Altman analysis is significant and systematic.

## 4 Discussion

The main results of this study are that 3D PREFUL MRI ventilation‐weighted measurements are (i) repeatable at both 3 and 1.5 T, (ii) consistent at 3 T, and (iii) show significant differences between 3‐ and 1.5‐T measurements. Repeatability assessments showed no significant bias between the first and second measurements, except for the mean of the RVent parameter at Center 2. These differences may be explained by the habituation effect, resulting in a more relaxed breathing pattern in subjects in Center 2 during the second measurement. This is supported by the 15% relative decrease in RVent values observed in the second measurement compared to the first. Another possible explanation may be the differences in the frequency of breathing between measurements [34], which might affect the static mean RVent values and the homogeneity of the breathing cycle. While no significant difference in breathing frequency was observed, the mean bias of −1.10 breaths/min suggests a slower breathing pattern during the second measurement, which may have resulted in a lower mean RVent parameter. CoV and ICC values reported here are in agreement with the current literature [14], underscoring substantial to almost perfect repeatability of the assessed parameters. The only exception was in FVL‐CM, which showed only fair repeatability at 1.5 T. A possible explanation is the lower spread of FVL‐CM values between subjects, which may have negatively affected the differentiation of subjects and, consequently, the overall agreement. Interestingly, at 3 T, the ICC and CoV values for these parameters improved and fell within the substantial to almost perfect repeatability range. For example, when examining the mean of FVL‐CM, increased interquartile range and CoV values were observed at 3 T compared to 1.5 T, indicating greater variability in the 3‐T data, which in turn improved the ICC values. While the ICC values of FVL‐CM were higher at 3 T, indicating better repeatability, the clinical use of FVL‐CM at 1.5 T would benefit from validation in patient cohorts to enhance the understanding of its implications for clinical reliability. In the regional repeatability comparison, the high spatial overlap values across scanners and centers (91%–97%) suggest strong agreement in the spatial distribution of ventilated and nonventilated regions. These results indicate that the VV (VV_RVent_ and VV_FVL‐CM_) maps are robust to changes in scanner type, supporting their potential use in multicenter studies.

The dual‐center comparisons at 3 T between both centers demonstrated a significant difference in the mean RVent parameter in the comparison of the second measurement. This finding is likely a result of the differences in tidal volumes between subjects at both centers. Importantly, no significant differences were seen in any of the other ventilation parameters, especially VVP, highlighting the consistency of the 3D PREFUL MRI technique. Furthermore, our results are consistent with other 3‐T studies that assessed dual‐site or multisite repeatability and reproducibility using other pulmonary MRI modalities, such as oxygen‐enhanced MRI in healthy volunteers [35] and hyperpolarized ^129^Xe MRI in people with cystic fibrosis [36, 37].

Significant differences between 3‐ and 1.5‐T ventilation‐weighted measurements at Center 1 were observed and were independent of tidal volume. These differences were further confirmed by effect size calculations, which indicated moderate to large effects for most of the 3D PREFUL MRI parameters. This suggests a systematic bias related to field strength rather than random error. The observed bias could potentially be reduced through calibration between the systems using phantom measurements or, more accurately, by correlating the ventilation‐weighted results to a gold standard ventilation measurement using hyperpolarized ^129^Xe or ^19^F techniques. However, such reference measurements were not available within the scope of this study. These findings highlight the importance of using scanners of similar field strengths in future trials. Comparing the observed differences to other imaging modalities, the VVP differences of 4%–5% seen in this study are slightly higher than the minimal clinically important difference of 2%–4% suggested for ventilation defect percentage values using ^3^He and ^129^Xe imaging [38, 39]. While the shift in VVP at 3 T towards higher values might be corrected using less‐conservative thresholds, the more pronounced heterogeneity of the raw ventilation‐weighted measurements at 3 T cannot be directly addressed. The differences in RVent reported here exceed 10% of the functional value, which was recently identified as the maximum absolute error for ventilation measurement in 2D self‐gated noncontrast‐enhanced functional lung MRI [40]. The most likely explanation for the differences observed between 3 and 1.5 T is the pronounced sensitivity of 3‐T data to susceptibility variations in lung parenchyma. From a technical perspective, 3‐T scanners should result in greater SNR compared to 1.5‐T scanners, with Gai et al. reporting a 1.8‐fold increase in SNR for 3 T using echo times of 0.09 and 0.14 ms [41]. However, due to the multiple air–tissue interfaces within the lung parenchyma, MR signal decays more rapidly at 3 T than at 1.5 T (T2* at 3 T is approximately 0.74 ms compared to 2.11 ms at 1.5 T) [16]. Even when using the stack‐of‐stars acquisition with a fully symmetrical echo and an echo time of approximately 0.8 ms as described here, much of the parenchymal signal may already be lost, potentially outweighing the theoretical advantage of 3 T in terms of improved SNR. Previous research reported significantly lower SNR in the lung at 3 T when compared to 1.5 T, which supports the observed inhomogeneities in ventilation parameters at 3 T [42]. Recently, Behrendt et al. demonstrated dependence of ventilation‐weighted parameters on TE at 1.5 T using 2D ultrashort echo time (UTE) acquisition [43]. Therefore, future research could focus on using UTE for functional ventilation techniques [13, 44] at 3 T to mitigate the faster signal decay observed at higher field strengths.

## 5 Limitation

The present study had several limitations. Firstly, only healthy subjects were recruited; therefore, the initial results should be elaborated in a future study with a larger cohort including patients with distinct pulmonary disease. Considering the exploratory nature of the study and the limited sample size, strict correction for multiple testing was avoided to reduce the potential for type II errors. Upcoming work should also evaluate the reproducibility of 3D PREFUL MRI on different vendor systems.

In the field strength comparison, true randomization was not achieved at Center 1 due to scheduled clinical routine scans. Specifically, 15 subjects underwent their first scan on the 1.5‐T scanner, with the remaining 9 on the 3‐T scanner. Although this is unlikely to have influenced the results, future repeatability studies should be designed with this in mind.

In the dual‐center comparison, two different scanners were used. While they both operated at 3 T, there were some technical differences in the gradient system performance (e.g., maximum gradient amplitude: 45 mT/m for MAGNETOM Skyra vs. 60 mT/m for MAGNETOM Vida) that may have influenced the results. We also acknowledge that sex was not matched in the dual‐center comparisons.

## 6 Conclusion

3D PREFUL MRI ventilation‐weighted parameters were highly repeatable at 1.5 and 3 T and consistent across two different centers at 3 T. The significant differences noted between data acquired using 3 T compared to 1.5 T likely reflect a systematic bias related to field strength‐dependent factors, such as magnetic susceptibility effects or differences in signal‐to‐noise ratio, and highlight the importance of using the same field strength in future multicenter trials. These results represent an important development in establishing the reliability of 3D PREFUL MRI for further applications, such as pulmonary disease monitoring.

## Conflicts of Interest

F.K., A.V., and J.V.‐C. are shareholders of BioVisioneers GmbH, a company with an interest in pulmonary magnetic resonance imaging methods. C.T.F. is the recipient of a Monash University postgraduate scholarship. R.G. is an employee of Siemens Healthineers AG, Erlangen, Germany. The remaining authors have no conflicts of interest to declare.

## Data Availability

The data that support the findings of this study are available on request from the corresponding author. The data are not publicly available due to privacy or ethical restrictions.

## References

[1] M. Deimling , V. Jellus , B. Geiger , and C. Chefd'hotel , “Time Resolved Lung Ventilation Imaging by Fourier Decomposition,” in Proceedings of the 16th Scientific Meeting of the International Society for Magnetic Resonance in Medicine (International Society for Magnetic Resonance in Medicine, 2008).

[2] G. Bauman , M. Puderbach , M. Deimling , et al., “Non‐Contrast‐Enhanced Perfusion and Ventilation Assessment of the Human Lung by Means of Fourier Decomposition in Proton MRI,” Magnetic Resonance in Medicine 62, no. 3 (2009): 656–664, 10.1002/mrm.22031. 19585597

[3] A. Voskrebenzev , M. Gutberlet , F. Klimeš , et al., “Feasibility of Quantitative Regional Ventilation and Perfusion Mapping With Phase‐Resolved Functional Lung (PREFUL) MRI in Healthy Volunteers and COPD, CTEPH, and CF Patients,” Magnetic Resonance in Medicine 79, no. 4 (2018): 2306–2314, 10.1002/mrm.26893. 28856715

[4] E. Ilicak , S. Ozdemir , J. Zapp , L. R. Schad , and F. G. Zöllner , “Dynamic Mode Decomposition of Dynamic MRI for Assessment of Pulmonary Ventilation and Perfusion,” Magnetic Resonance in Medicine 90, no. 2 (2023): 761–769, 10.1002/mrm.29656. 36989180

[5] Z. J. T. Peggs , J. P. Brooke , C. E. Bolton , I. P. Hall , S. T. Francis , and P. A. Gowland , “Free‐Breathing Functional Pulmonary Proton MRI: A Novel Approach Using Voxel‐Wise Lung Ventilation (VOLVE) Assessment in Healthy Volunteers and Patients With Chronic Obstructive Pulmonary Disease,” Journal of Magnetic Resonance Imaging 61 (2024): 663–675, 10.1002/jmri.29444. 38819593 PMC11706312

[6] L. Mendes Pereira , T. Wech , A. M. Weng , et al., “UTE‐SENCEFUL: First Results for 3D High‐Resolution Lung Ventilation Imaging,” Magnetic Resonance in Medicine 81, no. 4 (2018): 2464–2473, 10.1002/mrm.27576. 30393947

[7] T. Boucneau , B. Fernandez , P. Larson , L. Darrasse , and X. Maître , “3D Magnetic Resonance Spirometry,” Scientific Reports 10, no. 1 (2019): 9649, 10.1038/s41598-020-66202-7. PMC7295793 32541799

[8] F. Tan , X. Zhu , M. Chan , et al., “Motion‐Compensated Low‐Rank Reconstruction for Simultaneous Structural and Functional UTE Lung MRI,” Magnetic Resonance in Medicine 90, no. 3 (2023): 1101–1113, 10.1002/mrm.29703. 37158318 PMC10501714

[9] S. Lee , H. Y. Lee , J. Park , H. Kim , and J. Y. Park , “Assessment of Pulmonary Ventilation Using 3D Ventilation Flow Capacity‐Weighted and Ventilation‐Weighted Maps From 3D Ultrashort Echo Time (UTE) MRI,” Journal of Magnetic Resonance Imaging 60, no. 2 (2024): 483–494, 10.1002/jmri.29129. 37970646

[10] F. Klimeš , A. Voskrebenzev , M. Gutberlet , et al., “3D Phase‐Resolved Functional Lung Ventilation MR Imaging in Healthy Volunteers and Patients With Chronic Pulmonary Disease,” Magnetic Resonance in Medicine 85, no. 2 (2021): 912–925, 10.1002/mrm.28482. 32926451

[11] F. Klimeš , A. J. Obert , J. Scheller , et al., “Comparison of Free‐Breathing 3D Phase‐Resolved Functional Lung (PREFUL) MRI With Dynamic ^19^F Ventilation MRI in Patients With Obstructive Lung Disease and Healthy Volunteers,” Journal of Magnetic Resonance Imaging 60 (2024): 1416–1431, 10.1002/jmri.29221. 38214459

[12] F. Klimeš , A. L. Kern , A. Voskrebenzev , et al., “Free‐Breathing 3D Phase‐Resolved Functional Lung MRI vs Breath‐Hold Hyperpolarized ^129^Xe Ventilation MRI in Patients With Chronic Obstructive Pulmonary Disease and Healthy Volunteers,” European Radiology 35 (2024): 943–956, 10.1007/s00330-024-10893-3. 39060494 PMC11782336

[13] S. Munidasa , B. Zanette , M. P. Dumas , et al., “Comparison of 3D UTE Free‐Breathing Lung MRI With Hyperpolarized ^129^Xe MRI in Pediatric Cystic Fibrosis,” Magnetic Resonance in Medicine 93 (2024): 1–13, 10.1002/mrm.30299. PMC11604841 39285622

[14] F. Klimeš , A. Voskrebenzev , M. Gutberlet , et al., “Repeatability of Dynamic 3D Phase‐Resolved Functional Lung (PREFUL) Ventilation MR Imaging in Patients With Chronic Obstructive Pulmonary Disease and Healthy Volunteers,” Journal of Magnetic Resonance Imaging 54, no. 2 (2021): 618–629, 10.1002/jmri.27543. 33565215

[15] M. Lederlin and Y. Crémillieux , “Three‐Dimensional Assessment of Lung Tissue Density Using a Clinical Ultrashort Echo Time at 3 Tesla: A Feasibility Study in Healthy Subjects,” Journal of Magnetic Resonance Imaging 40, no. 4 (2014): 839–847, 10.1002/jmri.24429. 24123396

[16] J. Yu , Y. Xue , and H. K. Song , “Comparison of Lung T2* During Free‐Breathing at 1.5 T and 3.0 T With Ultrashort Echo Time Imaging,” Magnetic Resonance in Medicine 66, no. 1 (2011): 248–254, 10.1002/mrm.22829. 21695727 PMC3122137

[17] F. Klimeš , A. Voskrebenzev , F. Wacker , and J. Vogel‐Claussen , “Three‐Dimensional Phase Resolved Functional Lung Magnetic Resonance Imaging,” Journal of Visualized Experiments 2024, no. 208 (2024): 1–21, 10.3791/66385. 38975766

[18] S. Rosenzweig , H. C. M. Holme , and M. Uecker , “Simple Auto‐Calibrated Gradient Delay Estimation From Few Spokes Using Radial Intersections (RING),” Magnetic Resonance in Medicine 81, no. 3 (2019): 1898–1906, 10.1002/mrm.27506. 30489652

[19] M. Uecker , F. Ong , J. Tamir , et al., “Berkeley Advanced Reconstruction Toolbox,” Proceedings of the International Society for Magnetic Resonance in Medicine 23 (2015): 2486.

[20] A. Voskrebenzev , M. Gutberlet , T. F. Kaireit , F. Wacker , and J. Vogel‐Claussen , “Low‐Pass Imaging of Dynamic Acquisitions (LIDA) With a Group‐Oriented Registration (GOREG) for Proton MR Imaging of Lung Ventilation,” Magnetic Resonance in Medicine 78, no. 4 (2017): 1496–1505, 10.1002/mrm.26526. 27859552

[21] D. Forsberg , Fordanic/Image‐Registration (GitHub, 2021), https://github.com/fordanic/image‐registration.

[22] F. Klimeš , A. Voskrebenzev , M. Gutberlet , et al., “Free‐Breathing Quantification of Regional Ventilation Derived by Phase‐Resolved Functional Lung (PREFUL) MRI,” NMR in Biomedicine 32 (2019): e4088, 10.1002/nbm.4088. 30908743

[23] A. Voskrebenzev , F. Klimeš , M. Gutberlet , et al., “Imaging‐Based Spirometry in Chronic Obstructive Pulmonary Disease (COPD) Patients Using Phase Resolved Functional Lung Imaging (PREFUL),” in Proceedings of the International Society for Magnetic Resonance in Medicine, vol. 26 (International Society for Magnetic Resonance in Medicine, 2018): 1079, http://indexsmart.mirasmart.com/ISMRM2018/PDFfiles/1079.html.

[24] T. Moher Alsady , A. Voskrebenzev , M. Greer , et al., “MRI‐Derived Regional Flow‐Volume Loop Parameters Detect Early‐Stage Chronic Lung Allograft Dysfunction,” Journal of Magnetic Resonance Imaging 50, no. 6 (2019): 1873–1882, 10.1002/jmri.26799. 31134705

[25] C. Crisosto , A. Voskrebenzev , M. Gutberlet , et al., “Artificially‐Generated Consolidations and Balanced Augmentation Increase Performance of U‐Net for Lung Parenchyma Segmentation on MR Images,” PLoS ONE 18, no. 5 (2023): e0285378, 10.1371/journal.pone.0285378. 37159468 PMC10168553

[26] M. M. Wernz , A. Voskrebenzev , R. A. Müller , et al., “Feasibility, Repeatability, and Correlation to Lung Function of Phase‐Resolved Functional Lung (PREFUL) MRI‐Derived Pulmonary Artery Pulse Wave Velocity Measurements,” Journal of Magnetic Resonance Imaging 60 (2024): 2216–2228, 10.1002/jmri.29337. 38460124

[27] M. R. Miller , J. Hankinson , V. Brusasco , et al., “Standardisation of Spirometry,” European Respiratory Journal 26, no. 2 (2005): 319–338, 10.1183/09031936.05.00034805. 16055882

[28] P. H. Quanjer , S. Stanojevic , T. J. Cole , et al., “Multi‐Ethnic Reference Values for Spirometry for the 3‐95‐Yr Age Range: The Global Lung Function 2012 Equations,” European Respiratory Journal 40, no. 6 (2012): 1324–1343, 10.1183/09031936.00080312. 22743675 PMC3786581

[29] T. F. Kaireit , A. Kern , A. Voskrebenzev , et al., “Flow Volume Loop and Regional Ventilation Assessment Using Phase‐Resolved Functional Lung (PREFUL) MRI: Comparison With ^129^Xenon Ventilation MRI and Lung Function Testing,” Journal of Magnetic Resonance Imaging 53, no. 4 (2021): 1092–1105, 10.1002/jmri.27452. 33247456

[30] J. Vogel‐Claussen , T. F. Kaireit , A. Voskrebenzev , et al., “Phase‐Resolved Functional Lung (PREFUL) MRI–Derived Ventilation and Perfusion Parameters Predict Future Lung Transplant Loss,” Radiology 307 (2023): 4, 10.1148/radiol.221958. 37070996

[31] P. E. Shrout and J. L. Fleiss , “Intraclass Correlations: Uses in Assessing Rater Reliability,” Psychological Bulletin 86, no. 2 (1979): 420–428, 10.1037/0033-2909.86.2.420. 18839484

[32] A. J. Viera and J. M. Garrett , “Understanding Interobserver Agreement: The Kappa Statistic,” Family Medicine 37, no. 5 (2005): 360–363. 15883903

[33] S. S. Sawilowsky , “Very Large and Huge Effect Sizes,” Journal of Modern Applied Statistical Methods 8, no. 2 (2009): 597–599, 10.22237/jmasm/1257035100.

[34] M. Lederlin , G. Bauman , M. Eichinger , et al., “Functional MRI Using Fourier Decomposition of Lung Signal: Reproducibility of Ventilation‐ and Perfusion‐Weighted Imaging in Healthy Volunteers,” European Journal of Radiology 82, no. 6 (2013): 1015–1022, 10.1016/j.ejrad.2012.12.003. 23295084

[35] M. Kim , J. H. Naish , S. H. Needleman , et al., “Feasibility of Dynamic T2*‐Based Oxygen‐Enhanced Lung MRI at 3T,” Magnetic Resonance in Medicine 91, no. 3 (2024): 972–986, 10.1002/mrm.29914. 38013206 PMC10952203

[36] M. J. Couch , R. Thomen , N. Kanhere , et al., “A Two‐Center Analysis of Hyperpolarized ^129^Xe Lung MRI in Stable Pediatric Cystic Fibrosis: Potential as a Biomarker for Multi‐Site Trials,” Journal of Cystic Fibrosis 18, no. 5 (2019): 728–733, 10.1016/j.jcf.2019.03.005. 30922812 PMC7054852

[37] L. L. Walkup , D. J. Roach , J. W. Plummer , et al., “Same‐Day Repeatability and 28‐Day Reproducibility of Xenon MRI Ventilation in Children With Cystic Fibrosis in a Multi‐Site Trial,” Journal of Magnetic Resonance Imaging 61 (2024): 1664–1674, 10.1002/jmri.29605. 39257323 PMC11896927

[38] R. L. Eddy , S. Svenningsen , D. G. McCormack , and G. Parraga , “What Is the Minimal Clinically Important Difference for Helium‐3 Magnetic Resonance Imaging Ventilation Defects?,” European Respiratory Journal 51, no. 6 (2018): 8–11, 10.1183/13993003.00324-2018. 29650564

[39] M. J. McIntosh , A. Biancaniello , H. K. Kooner , et al., “ ^129^Xe MRI Ventilation Defects in Asthma: What Is the Upper Limit of Normal and Minimal Clinically Important Difference?,” Academic Radiology 30, no. 12 (2023): 3114–3123, 10.1016/j.acra.2023.03.010. 37032278

[40] A. Slawig , A. M. Weng , S. Veldhoen , and H. Köstler , “A Bootstrapping Residuals Approach to Determine the Error in Quantitative Functional Lung Imaging,” Magnetic Resonance in Medicine 93, no. 4 (2025): 1484–1498, 10.1002/mrm.30367. 39552181 PMC11782724

[41] N. D. Gai , A. Malayeri , H. Agarwal , R. Evers , and D. Bluemke , “Evaluation of Optimized Breath‐Hold and Free‐Breathing 3D Ultrashort Echo Time Contrast Agent‐Free MRI of the Human Lung,” Journal of Magnetic Resonance Imaging 43, no. 5 (2016): 1230–1238, 10.1002/jmri.25073. 26458867 PMC4833722

[42] G. Chassagnon , C. Martin , W. Ben Hassen , et al., “High‐Resolution Lung MRI With Ultrashort‐TE: 1.5 or 3 Tesla?,” Magnetic Resonance Imaging 61, no. March (2019): 97–103, 10.1016/j.mri.2019.04.015. 31051201

[43] L. Behrendt , M. Gutberlet , A. Voskrebenzev , et al., “Influence of Echo Time on Pulmonary Ventilation and Perfusion Derived by Phase‐Resolved Functional Lung (PREFUL) MRI Using Multi‐Echo Ultrashort Echo Time Acquisition,” NMR in Biomedicine 37 (2024): 1–16, 10.1002/nbm.5270. 39367655

[44] F. Klimeš , J. W. Plummer , M. M. Willmering , et al., “Quantifying Spatial and Dynamic Lung Abnormalities With 3D PREFUL FLORET UTE Imaging: A Feasibility Study,” Magnetic Resonance in Medicine 93, no. 5 (2025): 1984–1998, 10.1002/mrm.30416. 39825520 PMC11893037

